# Cervical Cancer Detection Techniques: A Chronological Review

**DOI:** 10.3390/diagnostics13101763

**Published:** 2023-05-17

**Authors:** Wan Azani Mustafa, Shahrina Ismail, Fahirah Syaliza Mokhtar, Hiam Alquran, Yazan Al-Issa

**Affiliations:** 1 Faculty of Electrical Engineering Technology, Campus Pauh Putra, Universiti Malaysia Perlis, Arau 02600, Perlis, Malaysia; 2 Advanced Computing (AdvComp), Centre of Excellence (CoE), Universiti Malaysia Perlis, Arau 02600, Perlis, Malaysia; 3 Faculty of Science and Technology, Universiti Sains Islam Malaysia (USIM), Bandar Baru Nilai 71800, Negeri Sembilan, Malaysia; 4 Faculty of Business, Economy and Social Development, Universiti Malaysia Terengganu, Kuala Nerus 21300, Terengganu, Malaysia; 5 Department of Biomedical Systems and Informatics Engineering, Yarmouk University, 556, Irbid 21163, Jordan; 6 Department of Computer Engineering, Yarmouk University, Irbid 22110, Jordan

**Keywords:** cervix, tumor, review, CAD

## Abstract

Cervical cancer is known as a major health problem globally, with high mortality as well as incidence rates. Over the years, there have been significant advancements in cervical cancer detection techniques, leading to improved accuracy, sensitivity, and specificity. This article provides a chronological review of cervical cancer detection techniques, from the traditional Pap smear test to the latest computer-aided detection (CAD) systems. The traditional method for cervical cancer screening is the Pap smear test. It consists of examining cervical cells under a microscope for abnormalities. However, this method is subjective and may miss precancerous lesions, leading to false negatives and a delayed diagnosis. Therefore, a growing interest has been in shown developing CAD methods to enhance cervical cancer screening. However, the effectiveness and reliability of CAD systems are still being evaluated. A systematic review of the literature was performed using the Scopus database to identify relevant studies on cervical cancer detection techniques published between 1996 and 2022. The search terms used included “(cervix OR cervical) AND (cancer OR tumor) AND (detect* OR diagnosis)”. Studies were included if they reported on the development or evaluation of cervical cancer detection techniques, including traditional methods and CAD systems. The results of the review showed that CAD technology for cervical cancer detection has come a long way since it was introduced in the 1990s. Early CAD systems utilized image processing and pattern recognition techniques to analyze digital images of cervical cells, with limited success due to low sensitivity and specificity. In the early 2000s, machine learning (ML) algorithms were introduced to the CAD field for cervical cancer detection, allowing for more accurate and automated analysis of digital images of cervical cells. ML-based CAD systems have shown promise in several studies, with improved sensitivity and specificity reported compared to traditional screening methods. In summary, this chronological review of cervical cancer detection techniques highlights the significant advancements made in this field over the past few decades. ML-based CAD systems have shown promise for improving the accuracy and sensitivity of cervical cancer detection. The Hybrid Intelligent System for Cervical Cancer Diagnosis (HISCCD) and the Automated Cervical Screening System (ACSS) are two of the most promising CAD systems. Still, deeper validation and research are required before being broadly accepted. Continued innovation and collaboration in this field may help enhance cervical cancer detection as well as ultimately reduce the disease’s burden on women worldwide.

## 1. Introduction

**Figure 1:**
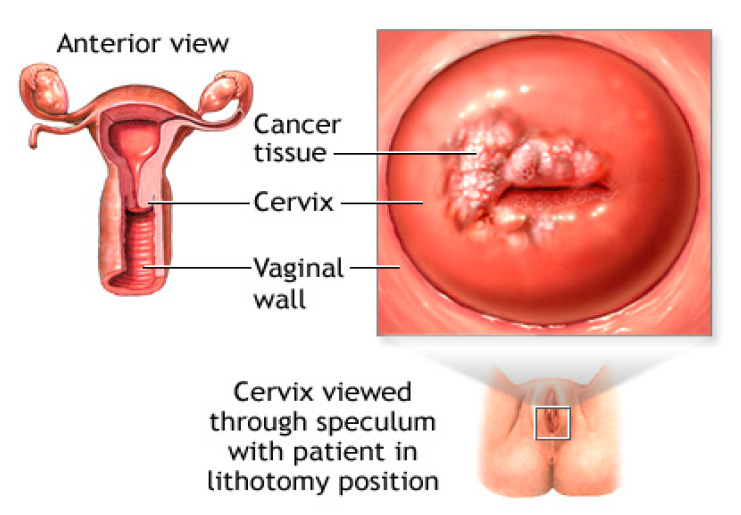
Female reproductive system [10].

In 2020, cervical cancer recorded 604,127 new cases and death in 341,831 cases, according to the Global Cancer Observatory (GCO) [1]. In Malaysia, cervical cancer is the fourth most common cancer among women, accounting for around 1740 newly diagnosed cases and 991 yearly fatalities in 2020 [2]. Every year, between 2000 and 3000 cases of cervical cancer are hospitalized in Malaysia, according to the Ministry of Health (MoH). The majority of these cases come late in the course of the disease. Malaysia’s mortality rate from cervical cancer is more than twice as high as that of the United Kingdom, the Netherlands, and Finland. The mortality rate has not decreased despite the implementation of screening programs and immunization campaigns against cervical cancer. The economic burden of cervical cancer is significant. In Malaysia, managing cervical cancer (from prevention to handling invasive diseases) costs around RM 312 million (USD 76 million). The majority of this (67%) goes towards treating aggressive cancer patients [3]. Pap smear screening is employed for early cervical cancer detection. The most crucial step is analyzing the Pap smear slide, and the identification of any condition or disease is crucial in order to administer the appropriate treatment [4,5]. Additionally, the Pap smear diagnostic reaction to a medication or treatment must be viewed or measured for clinical research. Clinically, microscope images are frequently utilized to diagnose Pap smear results. The sample images in the traditional approach, which involves taking a sample image under a microscope, run the risk of blurring effects, noise, shadows, lighting issues, as well as artifact issues on the images of thin smears [6,7]. Images from a Pap smear may have noise or other artifacts. Images from Pap smears may have poorer quality owing to noise or low contrast. Since the diagnosis relies on an individual, there are risks associated with the conventional procedure that might result in incorrect findings. A woman’s cervix is where cervical cancer first develops. The female reproductive system is depicted in Figure 1 [8]. It happens as a result of abnormal cervix cell growth [9]. The cervix and tissues nearby, as well as organs consisting of the liver or lungs, will be invaded by this. Human papillomavirus (HPV) infection is linked to an increased risk of generating abnormal cells. Abnormal menstruation, irregular menstruation, heavy menstruation, weight loss, pelvic pain, and vaginal discomfort are the initial indications of cervical cancer.

Cervical cancer is caused by a group of viruses called HPV. Having sexual activity with another person may transmit HPV. There is evidence that HPV plays a role in the occurrence of penis, vagina, vulva, and anus cancers. There are more than 100 types of HPV, and HPV types 16 and 18 account for approximately 70% of all cervical cancer cases globally [11]. All women ranging in age from 25 to 74 are invited to screening tests. There are various methods to screen the cervical lining using a colposcopy, which is used to magnify the area that the doctor wants to check after inserting the speculum into the vagina to check both the vagina and the cervix [12].

Early detection of cervical cancer is crucial since late diagnosis reduces the chance of survival in the entire world’s female population [13]. According to Logeswaran (2020), 90% of women with cervical cancer diagnoses in low- and middle-income countries such as India may die unexpectedly as a consequence of inadequate detection, early diagnosis, effective screening, and treatment [14]. J. Lu et al. (2020) conducted a similar study and discovered that early screening is the most successful strategy for reducing the worldwide cervical cancer burden. Nonetheless, because of a lack of information, limited access to medical facilities, and prohibitively costly processes in developing countries, vulnerable patient populations are unable to bear routine examinations [15].

It may be diagnosed using a variety of screening tests, but the Papanicolaou smear test, which employs cell cytology, is the most common. It is a reliable method for detecting cervical cancer, although there is always a possibility of misinterpretation owing to human observational mistakes [16,17]. According to a study conducted in the medical field by Jaya and Latha (2019), image processing plays a crucial role in making the correct choice by utilizing a variety of techniques and algorithms. However, it is difficult to detect Pap smear images through microscopes. Traditional cervical cell screening also relies heavily on the pathologists’ experience, which has the disadvantages of poor efficiency as well as low accuracy. Cervical cancer cells do not differ much in texture or color from normal cells, making their detection with smear tests very difficult [18].

However, cone biopsy screening is used when an abnormal cell is suspected in the cervix in order to detect it early. The most common screen test, as well as the Pap smear, also called the Papanicolaou test, is based mainly on using a brush to remove a small part of the lining tissue and checking it under microscopic levels to see if there are changes in the cell. This type of test can be used to discover if there is an infection or inflammation in the cervix or the presence of the HPV virus. The resultant images that have been obtained are called Pap smear images, which form a huge factor in early cervical cancer detection as well as classification. The new method for screening is based on the detection of HPV absence or presence [19]. Much research is carried out on the detection and classification of this type of cancer utilizing nanotechnology and building a biosensor to detect HPV, as well as using Pap smear images to detect and classify abnormal cells utilizing the benefits of deep and machine learning (ML) techniques. Other research focused on electrical impedance matching of affected signals with a 3D finite element model for cancer and non-cancerous cells. Cervical cancer affects the female reproductive system and is strongly associated with HPV infection, obesity, smoking, and sexually transmitted diseases (STDs). Manual Pap tests (Papanicolaou tests) are widely used for the early detection of cancer, but they are costly, stagnant, and highly dependent on the pathologist’s expertise. Several computer aided diagnostic (CAD) systems were developed to automatically detect cervical cancer. Developing automatic prediction models to identify vulnerable patients can improve the efficacy of screening programs and eliminate inconsistencies and subjectivity resulting from cytopathologists’ lack of expertise.

## 2. Materials and Methods

The primary goal of this study is to explore and understand the methodology of cervical cancer detection around the world between 1996 and 2022. The purpose of the current narrative analysis is to respond to the primary research question: (1) What types of cervical cancer detection have been proposed around the world? (2) How effective were computer-aided diagnostics for the Pap smear screening process? Contrary to that, cervical cancer detection has evolved significantly over the years, with several different techniques now available. The Pap smear test remains the most frequently employed method. Still, newer techniques such as visual inspection with acetic acid (VIA) and HPV testing, as well as Lugol’s iodine, are becoming more widely used. Early detection is the key to successful treatment and improved outcomes, and women should undergo regular cervical cancer screening according to recommended guidelines. In addition, this part discusses the requirement for a comprehensive evaluation of the cervical cancer situation. The outline of this review paper consists of three sections: Section 1 discusses an introduction and related research, and Section 2 describes the review data. The conclusions of this research are discussed in Section 3.

A method for obtaining the literature is shown in Table 1. The systematic review approach comprises three primary phases that were employed to determine the many relevant publications for this study. The initial phase is keyword recognition and the search for connected, related phrases utilizing the encyclopedia, dictionaries, and thesaurus, as well as prior research. Therefore, search strings were developed for the Scopus database once all pertinent terms were chosen. Considering literature (research papers) is the main source of pertinent information, it was the initial criterion. It also covers the exclusion of conference proceedings, chapters, books, book series, meta-synthesis, meta-analysis, reviews, and systematic reviews from the present research. Additionally, the review was limited to English-language studies only. A total of 108 publications were chosen in accordance with particular parameters.

**Figure 2:**
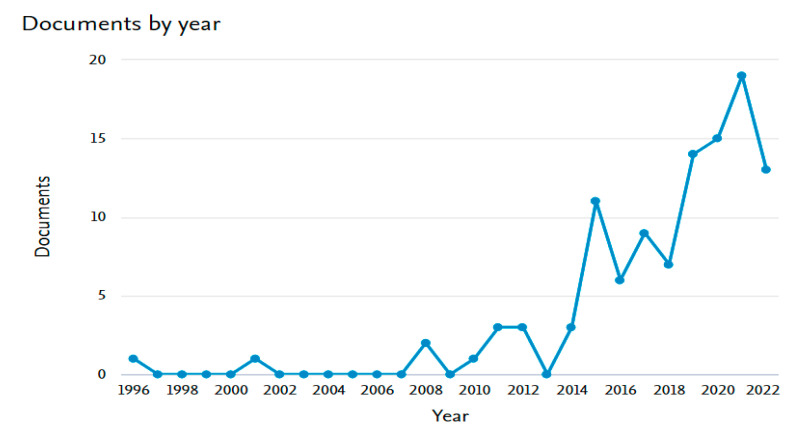
Number of documents per year.

Figure 2 represents the number of documents about cervical cancer per year. Obviously, interest in this topic started in 1996 with only one paper, and no production from 1997 to 2001 appeared in other documents. The settlement of ignorance was shown from 2002 to 2007, and two documents appeared in 2008. The steady increasing pattern appeared from 2009 to 2011. The sharp growth appeared from 2009 to 2015. In 2015–2018, there were swings between increasing and decreasing, but the average number was around eight documents per year. The total number sharply increased from 2018 to 2022 to be the mean of around 15 documents per year as well. That reflects people’s consideration of the danger of cervical cancer as well as the significance of research to build a solid understanding of the nature of the disease and the tools to overcome or reduce its impacts on women.

## 3. Review of the Study

### 3.1. 1996–2015

Many studies have been conducted in the past to investigate cervical cancer diagnosis. Worldwide research is being conducted by doctors to better understand cervical cancer, how to prevent it, how to cure it, and how to provide treatment for those who have been diagnosed with the disease. For example, in 1996, an innovative method for the creation of segmentation and diagnostic algorithms for biomedical image analysis was given by [20]. In this case, a prototype expert system was created to give gynecologists a reliable and objective tool. Moreover, a collection of knowledge sources was created using specialized image-analysis methods. The robust control method employed by the expert system reduces the need for domain-specific control knowledge and has been shown to efficiently identify cervical cancer. The composition of segmentation and diagnostic methods for biomedical image analysis was also discussed in this paper, employing a new technique.

After many years, cervical cancer diagnosis evolved due to technological development. Following that, in 2001 [21], it was stated that the principal component analysis (PCA) in the wavelet domain delivers robust novel features with regard to the non-invasive detection of cervical intraepithelial neoplasia (CIN) employing fluorescence imaging spectroscopy. The term “principal wavelet components” (PWCs) refers to these characteristics. Average accurate classification rates for five cervical tissue classes—low-grade dysplasia (CIN 1), squamous, columnar, and metaplasia—as well as a fifth class for other unidentified tissue types, blood, and mucus—were 95% when PWC characteristics were employed as inputs to a 5-class NN. Apart from these [22], we presented a new technique to determine cervical cancer employing microwaves to measure the dielectric properties of the smear at microwave frequencies. This measuring approach is easy, and the smear collection is non-surgical and painless. The findings propose another option to the Papanikolaou or Papanicolaou tests and demonstrate a new technique for detecting cervical cancer using microwave measurement that may offer a less invasive alternative to these surgical procedures for detecting the disease.

On the other hand, in vivo, cervical dysplasia and cancer detection utilizing model-based analysis of reflectance and fluorescence spectra have been proven [23]. Here, a theory-based diffusion model is employed along with two analytical methods for calculating reflectance spectra that are contrasted with Monte Carlo simulations. A diagnostic algorithm is also created and tested utilizing cross-validation based on these obtained parameters. This algorithm’s sensitivity/specificity for each measurement in comparison to the gold standard of histopathology are 85/51%. The accuracy described in previous research using optical technology to identify cervical cancer and its precursors corresponds to this.

Meanwhile, in [24], a quantitative colposcopic imaging system for early cervical cancer diagnosis is assessed in a clinical study. The cervix of living human beings is employed to assess the kinetics of the acetowhitening process in order to obtain diagnostic information. The imaging method relies on 3D active stereo vision as well as motion tracking. It was possible to distinguish between normal tissue and HPV-infected tissue, as well as low-grade and high-grade CIN lesions, utilizing a diagnostic algorithm with 91% SE and 90% SP. The findings show that the quantitative colposcopic imaging system may be able to deliver unbiased screening and diagnostic information for the early detection of cervical cancer.

Additionally, [25] immobilized anti-HPV18 and *E. coli* O157: H7 antibodies on magnetic silica-coated Fe_3_O_4_ for early diagnosis of cervical cancer as well as diarrhea. Uncoated Fe_3_O_4_ nanoparticles having a 9–16 nm average diameter as well as a saturation magnetization of around 66 emu/g were first prepared using the co-precipitation method. The findings revealed that magnetic SiO_2_-coated Fe_3_O_4_ nanoparticles could be an auspicious contender for diagnosing cervical cancer at an early stage, specifically with high accuracy.

In 2011, [26] employed an optoelectronic method to detect CIN as well as cervical cancer. The pNOR number and the sensitivity/specificity of the optoelectronic approach were shown by the authors to be correlated. The specificity of the optoelectronic approach was calculated to be 65.70% for LGSIL and 90.38% for HGSIL and cervix squamous cell carcinoma. The optoelectronic technique utilized to validate the absence of cervical pathology was assessed to have a 78.89% specificity. Here, CIN, which exists in the squamous epithelium as well as squamous cell carcinoma of the cervix, is easily detected using the optoelectronic approach.

In the same year, [27] investigated the hWAPL histological expression value assessment in the cytological as well as histological diagnosis with regard to cervical intraepithelial neoplasia and cervical cancer. The expression intensity of hWAPL protein in the HSIL group, LSIL group, ASCUS group, and ASC-H group was obviously greater than that in the NILM group (*p* < 0.05), and the expression intensity in the ASCUS group and ASC-H group was higher than that in the LSIL group (*p* < 0.05). Furthermore, in the ASCUS and ASC-H groups, the frequency of SCC + CIN III was above 50%. Therefore, hWAPL may be a promising candidate for diagnosing low-grade CIN. Furthermore, the histological expression of hWAPL is consistent with the cervical lesions’ cytological type.

A year later, in 2012, in order to enhance cervical cancer risk classification, [28] investigated the automated detection of dual p16/Ki67 nuclear immunoreactivity in liquid-based Pap tests. Algorithms were created to digitize and examine smears stained with p16 as well as Ki67 antibodies. The nuclear mask was produced employing a gradient-based radial symmetry operator along with adaptive symmetry image processing. This was subsequently followed by the extraction of attributes from each nucleus, such as pixel data as well as immunoreactivity signatures. The quantitative analysis of immunoreactivity offered by the further emphasis on classified nuclei, according to the authors, may have a positive influence on the effectiveness and screening results of the Pap test.

In the same year, which is 2012 [29], a new technique was proposed to construct a tumor probability map while gradually determining the boundaries of an organ of interest on the basis of the accomplished nonrigid transformation. The technique dealt with the difficulties of considerable tumor regression and its impact on nearby tissues. Findings indicate that the suggested technique greatly surpasses the current registration algorithms and reaches a precision equivalent to manual segmentation. Additionally, there is excellent agreement between the suggested method’s tumor detection results and manual delineation by an experienced doctor.

Moreover, in [30], blood and urine samples from cervical cancer patients were collected, and their fluorescence emission spectra (FES) as well as Stokes shift spectra (SSS) were contrasted to those of normal controls. Both spectra demonstrated that in cervical cancer patients, the relative levels of biomolecules, which include flavin, nicotinamide, adenine dinucleotide, collagen, and porphyrin, were out of balance. The author also stated that this is the first study on FES and SSS of blood and urine samples from patients with cervical cancer that provides a sensitivity of 80% as well as a specificity of 78%.

A total of 2 years later, in 2014 [31], it was proposed to use time-resolved blood component spectra to identify cervical cancer. Porphyrin served as the biomarker indicative of cancer in this instance, with samples from cancer patients having fluorescence decay times that are 60% greater than those from normal controls. A randomized set of samples from cancer patients and controls (*n* = 27 in total) could be categorized with sensitivity (92%) and specificity (86%) using these parameters.

Utilizing reduced graphene oxide–tetraethylene pentamine as electrode materials and distinct redox probes as labels [32], this suggested simultaneous electrochemical detection of cervical cancer indicators in the same year. In accordance with the peak current change of neutral red and thionine prior to and following the antigen-antibody reaction, the immunosensor was constructed with a sandwich structure. According to the findings, the immunosensor exhibited a broad linear range, a small detection limit, high reproducibility, and stability. Furthermore, the technique has been employed successfully to examine serum samples.

Moreover, [33] utilized extracted intrinsic fluorescence as well as PCA to identify the advancement of cervical cancer. Here, along with the intrinsic fluorescence, the effectiveness of PCA in separating the aggregate behavior from smaller associated clusters in a dimensionally diminished space is tested. By closely observing the sectorial behavior of the dominant eigenvectors of PCA, it is possible to determine the various activities of the dominant fluorophores, flavins, nicotinamide adenine dinucleotide, collagen, and porphyrin of various classes of precancers. The Mahalanobis distance was also computed utilizing the scores of the chosen major components in order to better categorize the various grades.

A year later, [34] presented a method for colposcopic images-based automated cervical cancer diagnosis. Here, abnormal and normal tissue are distinguished using wavelet and statistically based attributes. The wavelet-decomposed image is employed to obtain the wavelet energies. The feature vector produced from the combination of these features is then applied to the detection. The segmented cancer region demonstrates that the suggested fusion technique is capable of identifying the cancer-affected region with more accuracy over the wavelet, along with statistical features-based approaches.

In addition, a hybrid classifier-based computer-aided detection (CAD) of cervical cancer utilizing Pap smear images was also suggested by [35] in 2015. It is utilized to divide the cell image from the test Pap smear into normal and dysplastic cell images. Following that, morphological techniques are employed to identify and segment the abnormal cell region. On images from databases with free access to the public, the suggested technique is evaluated. A unique illumination correction and intensity normalization approach on cervigrams was put out by [36] in the same year in order to aid in the early detection of uterine cervical cancer. In light of our study’s results, we draw the conclusion that the peak of the squamous epithelium (SE) region’s intensity distribution and the peak of the entire cervix region are significantly associated.

Furthermore, by using the nested structure of its data to extract patient-level features from the cell-level data, utilizing a statistical model that takes advantage of the hierarchical data structure, and classifying the cellular level [37], it executed comparative research on three primary methods for solving problems. With an estimated 61% sensitivity and 89% specificity on independent data, the optimal method was to classify at the cellular level and count the number of cells with a posterior probability larger than a threshold value. In addition, recent advancements in statistical learning make it feasible to reach great accuracy. Apart from that, new clinical studies that support the use of HPV E6/E7 mRNA as a marker in advanced cervical cancer screening programs were reported in 2015 [38]. The authors give a general review of the research study sample size, age, recruitment setting, HPV mRNA, and HPV DNA tests. It was demonstrated by the pooled evaluation of clinical research that HPV mRNA may be a useful diagnostic biomarker. To draw a firm conclusion, however, further research must be conducted.

On the other hand, in the same year, [39] investigated the degree of squaraine dye aggregation that affects the strength of surface-enhanced Raman signal scattering (SERS) after adsorption on a gold surface that has been nano-roughened. When chemisorbed on spherical gold nanoparticles, the SQ2 (mono lipoic acid appended), SQ5 (conjugated with hexyl and dodecyl side chains), and SQ6 (conjugated with hexyl and dodecyl side chains) squaraine derivatives demonstrated a substantial rise in Raman scattering in the fingerprint region. HeLa cells demonstrated pronounced SERS mapping intensity and selectivity towards the cell surface and nucleus after further conjugating this nanotag with monoclonal antibodies that targeted overexpressed receptors, EGFR and p16/Ki-67, in cervical cancer cells.

Subsequently, [40] proposes a system for automatically classifying and segmenting cervical cells. Radiating Gradient Vector Flow (RGVF) Snake is employed to separate the cytoplasm, nucleus, and background of a single cervical cell image. For system training, several cellular and nuclear properties are retrieved. Artificial neural networks (ANN) are employed to examine the dataset’s ability to categorize seven distinct cell types and distinguish between abnormal and normal cells. The clinical research on styping identification of HPV infection using microarrays from paraffin-embedded tissues of precursor lesions as well as cervical cancer was also explored by [41]. This led to the identification of the prevalence and type distribution of HPV in cervical cancer and CIN in Jiangsu, China. The findings indicate that Jiangsu’s (China’s) high rate of HPV 16, 18, 33, 31, and 58 warrants further notice. It has significant repercussions for the effective administration of the HPV vaccination and the selection of testing techniques.

Apart from that, [42] examined the fractal dimension of AFM images of human cervical epithelial cells at various stages of cancer growth to evaluate the early detection of cervical cancer. Individual human cervical epithelial cells at three phases of cancer progression—normal, immortal (pre-malignant), and carcinoma cells—were examined using the AFM HarmoniX modality by the author. The authors were successful in distinguishing between abnormal and normal cells by utilizing AFM to examine the surface characteristics of human cervical epithelial cells. This technique could supplement current techniques to improve the accuracy of diagnosis.

Moreover, [43] proposed using nanotechnology and biomarkers for cervical cancer’s early detection and treatment. Nanomaterials are special in their optical, physical, and electrical characteristics, which has made them particularly advantageous for sensing. Cancer biomarkers, which are employed as targets in the detection and monitoring of cancer, are mostly composed of RNA fragments, DNA fragments, antibody fragments, and proteins. In a few decades, it is expected to be feasible to identify cancer at a very early stage, giving a significantly greater probability of treatment.

Subsequently, [44] describes an ultrasensitive electrochemical immunosensor for accurate detection of p16 and shows how effectively it performs when used with patient cell lysates to detect solubilized p16 protein. Furthermore, the authors also reported that the suggested immunosensor successfully detected raised p16 levels in cervical swab samples taken from 10 patients who had received positive results from a standard Pap smear test, demonstrating that electrochemical immunosensors hold great potential for the early detection of cervical cancer in a clinical setting.

### 3.2. 2016–2018

Several studies tried to diagnose cervical cancer using various techniques. For instance, in 2015, Yulan Wang et al. recommended the use of fluorescence lifetime imaging microscopy (FLIM) for the early detection of cervical cancer. They discovered that the lifetime of cancerous cells was shorter compared to normal cells. They recommend FLIM as a highly precise and specific method that can detect the occurrence of precancerous as well as cancerous cells quickly [45].

In 2016, S. Athinarayanan et al. [46] suggested an automatic multistage cervical cancer diagnostic system using Pap smear images (obtained from the Herlev dataset described in Table 2) and machine learning (ML) methods. In the preprocessing stage, images were denoised, intensity and texture features were extracted, and finally, images were differentiated using SVM into normal and abnormal classes. They succeeded in detecting cervical cancer with 94% accuracy. Moreover, Anousouya Devi et al. [47] developed an image analysis algorithm to replace time-consuming Pap smear screening tests. The authors discussed a variety of segmentation algorithms and feature extraction techniques with regard to the efficient segmentation of Pap smear slides.

Furthermore, Xianfeng Xu et al. [48] investigated the value of PET/CT scanning in detecting cervical carcinoma in 51 patients. Note that PET/CT diagnosis capability is superior to the classical FIGO discrimination technique. For example, PET/CT detected primary tumors with 84.31% accuracy, 80.77% specificity, and 88% sensitivity. On the other hand, it detected lymph nodes with 76.47% accuracy, 71.43% specificity, and 82.61% sensitivity. Subsequently, Jose Amaya et al. [49] designed a high-stability voltage current source for the recognition of cervical cancer using electrical bio-impedance spectroscopy. Here, the medical kit they designed was compatible with international standards. Finally, Rizanda Sobar et al. [50] determined seven behavior features and surveyed 72 respondents (including 22 cancer patients) in Indonesia. They used two machine learning (ML) techniques, particularly logistic regression (LR) and Naïve Bayes, to forecast the risk of becoming a cervical cancer patient. With respect to accuracy, Naïve Bayes outperformed LR (91.67% compared to 87.5%), and with respect to AUC, LR outperformed Naïve Bayes (0.97 compared to 0.96).

One year later, Irvin Sitompul et al. [51] conducted a descriptive qualitative study using a questionnaire to evaluate the knowledge of aged women in the Cakung health center regarding the early detection and prevention of cervical cancer. They concluded that knowledge of the Human Papilloma Virus (HPV) vaccine is weak. Meanwhile, Branislava Jeftic et al. [52] presented a cervical cancer detection method relying on optomagnetic imaging spectroscopy (OMIS) and compared the findings utilizing unstained and stained Papanicolaou smears. Using the Naïve Bayes classifier, they separated the samples into four groups: the II Pap group (normal cells), the III Pap group (abnormal cells), and the IV and V Pap group (cancerous cells). Unstained sample classification with Naïve Bayes achieved 96% accuracy, whereas stained sample classification achieved 85.18% accuracy. Apart from these, Abdullah Iliyasu et al. [53] proposed a quantum hybrid technique that uses quantum particle swarm optimization (QPSO) for selecting 7 out of 17 features, as well as a fuzzy KNN for the classification of cervical cells in smeared images. They used 917 images from the Herlev dataset and achieved 86% recall, 85% precision, and F1 score of 85%. On the other hand, Wen Wu et al. [54] employed three SVM-based combinations for the diagnosis of cervical cancer. All four target variables were identified, and the performance of SVM was superior to SVM-RFE and SVM-PCA. SVM achieved high precision using all 30 features, but the computation cost was high. The authors showed that the SVM-RFE and SVM-PCA gave comparable performance to the SVM using only 8 features, improving classification time considerably.

In the same year, Katrin Carow et al. [55] presented evidence that the incorporation of HPV-DNA into the host genome is an initial step in the formation of cervical cancer. They recommend using viral-cellular junction sites as biomarkers when examining circulating tumors. Meanwhile, Vidya Kudva et al. [56] proposed an image-processing approach that can be used as an image treatment step in any cervix cancer detection system. They presented a cervix region segmentation method and detected specular reflections with high precision, irrespective of lighting conditions and color variations. Apart from these, Guanglu Sun et al. [57] suggested an ML framework relying on relief feature selection and a Random Forest (RF) classifier to diagnose cervical cancer. They used 917 Pap smear images obtained from the Herlev dataset together with 10-fold cross-validation to perform binary classification. RF outperformed LR, C4.5, and Naïve Bayes classifiers with 94.44% accuracy and 0.9804 AUC using 13 features. In addition, Rubina Shaikh et al. [58] compared two optical modalities, particularly Raman (RS) and Diffuse Reflectance Spectroscopy (DRS), in differentiating between normal and abnormal cells. One hundred forty-six recorded spectra (67 tumors and 79 normal) were analyzed using a combination of Principal Component and Linear Discriminant Analysis ML techniques. They used Leave One Out Cross Validation (LOOCV) and concluded that DRS is more suited for rural areas, whereas RS is suited for developing countries. Furthermore, Muljo et al. developed an online learning management prototype to educate health workers and the public in Indonesia about early cervical cancer detection as well as treatment [59].

In 2018, Mithlesh Arya et al. [60] used SVM as well as ANN to classify single-cell images captured from Pap smear slides into benign and malignant tumors. The accuracy obtained using the suggested texture-based features exceeds that obtained using shape-based features. Additionally, the performance obtained using a combination of features was better than that obtained using a single feature. Using quadratic SVM, they achieved 99.5% accuracy, 99% sensitivity, and 99% specificity. Meanwhile, Ashutosh Sharma et al. [61] successfully employed fluoranthene-based yellow fluorescent lipid probes with respect to the detection of lipid droplets in cervical cancer tissues. FLUN-550 and FLUN-552 quantitatively detected the excess lipid accumulation and were really useful in the early diagnosis of human cervical cancer. Additionally, Kelwin Fernandes et al. [62] developed a supervised deep learning (DL) method to diagnose cervical cancer with high accuracy using the medical records of 858 patients. To study the impact of their architecture, they applied their methodology to different datasets and demonstrated that their efficiency is not limited to cervical cancer. They used a loss function for dimensionality reduction, achieving an AUC of 0.6875. Furthermore, Yueyue Jing et al. [63] established quick, highly sensitive, and highly specific label-free imaging and spectroscopy for the detection of cervical tumors compared to the traditional clinical staining method. They studied unstained tissues extracted from 38 patients and achieved 100% sensitivity and 91% specificity.

In the same year, Rocky Dillak et al. [64] suggested an early alarm system to diagnose cervical cancer based on a combination of chaos optimization and ridge polynomial neural networks. They achieved an accuracy of 96%, a sensitivity of 95.56%, and a specificity of 96.67%. Apart from these, Vidya Kudva et al. [65] manually extracted 102 images obtained during visual inspection with acetic acid; 42 images were pathologic, and the remaining 60 were negative. They used a shallow-layer CNN to discriminate between cancer and non-cancer lesions by automatically extracting features from 684 representative patches with 100% accuracy. Following that, Sherif Abdoh et al. [66] identified 32 risk factors to build a cervical cancer diagnosis framework. They employed two feature reduction techniques, namely Recursive Feature Elimination (RFE) and PCA. Furthermore, they used an RF classifier combined with the Synthetic Minority Oversampling Technique (SMOTE) to correctly classify cervical cancers. The obtained results were validated using 10-fold cross-validation, and SMOTE-RF outperformed SMOTE-RF-RFE and SMOTE-RF-PCA in detecting all 4 cancer groups.

### 3.3. 2019–2020

As artificial intelligence (AI) and image processing technology advance, we have reviewed progressively intelligent diagnosis tools that are being applied in cervical cancer screening. In this section, we offer a brief review of some methods available in the literature, starting with the year 2019 and progressing to the current cervical screening. Lavanya Devi et al. (2019), for instance, investigate the various automated methods for detecting abnormal cells in Pap images. Cancer screening commonly includes a Pap smear test and an acetic acid test. Cells from the vagina and cervix are extracted and analyzed under a microscope for the occurrence of an abnormal cell in a pap test. An acetic acid test is employed to identify the existence of abnormal cells by comparing the differences in characteristics between samples before and after the application of acetic acid. According to the report, automated screening has become more common than manual screening, given that the latter is inaccurate [67]. This method of screening has been endorsed in a study conducted by Abdullah et al. (2019), where computer-based algorithms are broadly employed in cervical cancer screening. In this research, a better cellular neural network (CNN) algorithm has been set up as a potential means of detecting cancerous cells in Pap smear images in real-time. For automated detection of cancerous cervix cells, a CNN built-in in MATLAB using templates that segment cell nuclei has been established. The simulation findings demonstrate that our suggested CNN algorithm can automatically identify cervix cancer cells with over 88% accuracy [68].

Jaya and Latha (2019) introduced a technique for enhancing Pap smear images by comparing Power Law Transformation for Gamma Correction, Histogram Equalization in the Contrast Stretching algorithm, Contrast Limited Adaptive Histogram Equalization (CLAHE), and Shading Correction. To determine the performance of upgraded Pap smear images, the quality measurement NAC, SC, PSNR, and MSE values were determined. As a programming tool, MATLAB R2016a and ANN classification were used to assess the accuracy level of each feature extraction of the algorithm. The study concluded that CLAHE produced a decent result for enhancement, and the SGLDM feature extraction algorithm achieved 93% accuracy while utilizing ANN [69]. A review of the literature undertaken found that accurate recognition of cervical cancer cells is crucial for clinical diagnosis. A better approach built around the residual neural network is presented to increase the accuracy of diagnosis. However, these current algorithms are only enhanced by the use of low-level manual features. The findings of the experiments demonstrate that the lightweight deep model performs better than the current comparative models and may obtain a model accuracy of 94.1% when applied to the cervical cell data set [70]. Hence, as recommended by William et al. (2019), it is advantageous to construct a computer-assisted diagnostic tool to increase the accuracy and reliability of the Pap smear test. In this research, Pap smear image analysis was utilized to construct a tool for the automated detection and classification of cervical cancer. Scene segmentation was accomplished using a trainable Weka segmentation classifier, while a sequential elimination strategy was employed for debris rejection. While classification was accomplished utilizing a fuzzy C-means technique, feature selection was accomplished employing simulated annealing combined with a wrapper filter [71]. The research found that three distinct datasets—single-cell images, multiple-cell images, and Pap smear slide images from a pathology lab—were utilized to evaluate the classifier. For each dataset, overall classification accuracy, sensitivity, and specificity results of “98.88%, 99.28% and 97.47%”, “97.64%, 98.08% and 97.16%”, and “95.00%, 100% and 90.00%”, accordingly, were attained. In comparison to the manual analysis, which takes between 5 and 10 min per slide, the suggested system can analyze a whole Pap smear slide in about 3 min.

Ref. [72] identified the relevant features in the cancer classification as well as optimized the model. The vital properties in the attribute list were explored using the binary cuckoo search optimization technique. The experimental findings demonstrate the greater performance of the Decision Tree (DT) classifier over all other classifiers, with accuracy increasing from 94.7% to 97% following cuckoo optimization. Another study conducted by Adem et al. (2019) discovered that softmax classification with a stacked autoencoder model, which was implemented for the first time in the cervical cancer dataset, performed better compared to other ML methods with an appropriate 97.8% classification rate. New techniques of diagnosis are described in this article in terms of patient diagnostic support systems, taking into account the interest in ML approaches in cancer research [73].

In the year 2020, a number of studies offered new screening methods, such as the Shot multiBox detector, which can accurately detect many items of multiple scales at the same time to solve the classic saliency cervical cancer diagnosis approach in ultrasound images. The study provides a new multi-saliency object detection model with an appended deconvolution module embedded within the residual attention module. Experiments demonstrate that the suggested diagnosis method beats comparable algorithms in terms of detection accuracy. It also improves the accuracy of cervical diagnosis by increasing detection performance for multi-saliency cervical cancer objects with small scales [74]. The Enhanced Johnson’s Algorithm (EJA) was proposed by Ali et al. (2019) as the new shortest path for detecting cervical cancer-associated genes in the Protein-to-Protein Interaction (PPI) network for early cervical cancer diagnosis in their study. EJA was also adopted to find the shortest path between invasive and pre-invasive genes. The Bellman-Ford approach was used in EJA to reconstruct the path with a new iterative matrix, which successfully reduced the elapsed time by omitting the negative cycles in the gene connection [75]. Huang et al. (2019) discovered that endogenous fluorophores in cells and tissues, such as diminished nicotinamide adenine dinucleotide (phosphate) (NAD(P)H) as well as flavin adenine dinucleotide (FAD), may be imaged by FLIM to illustrate the tissue morphology features, including the biomolecular variations in the microenvironment. It was shown that by monitoring the fluorescence lifetime of NAD(P)H as well as FAD in nearby healthy cervical tissues, benign uterine tumors with abnormal cell development, which include leiomyomas and adenomyosis, may be identified [76]. According to [18], cervical cancer is caused by morphological alterations in cells or dead nuclei in the cervix. The detection of abnormalities in cells necessitated a high-level digital image processing technique that included an automated, complete ML skill set. To split the cytoplasm as well as the nucleus from the cell, an innovative fuzzy-based approach has been proposed. KNN is instructed with the color and form attributes of the segmented cell units, and then it is used to classify unknown cervix cell samples. The cytoplasm, as well as the nucleus of the cervix cell, are given shape and color using the proposed technique.

Several other methods have been introduced in detecting this disease, such as automatic feature extraction and classification for acetic acid and Lugol’s iodine cervigrams, as well as (2) methods for merging diagnosis/features of distinct contrasts in cervigrams for enhanced performance, which attained a sensitivity, specificity, and accuracy of 81.3%, 78.6%, and 80.0%, respectively [77]. A study reported that a novel immunosensor had been formed for quantitative detection with respect to the squamous cell carcinoma antigen (SCCA) in cervical cancer, built on surface-enhanced Raman scattering (SERS). The SCCA monoclonal antibody was combined with polydopamine resin microspheres covered with gold nanoparticles as capture substrates. Phosphate buffer (PBS) had a detection limit of 7.16 pg mL^−1^ and human peripheral blood had a detection limit of 8.03 pg LH^−1^. The findings showed that the SERS immunoassay approach has a possibility for use in early cervical cancer screening and diagnosis [78]. Fuzzy Swallow Swarm Based Feature Selection (FSSBFS) has been introduced for the optimal selection of cervical cancer features. The proposed ISVM-FssBFS classifier is improved when compared to SVM and Multilayer Perception Classifier (MLP) classifiers. The cervical cancer samples are characterized by 32 risk factors and four target classes: Biopsy, Cytology, Schiller, and Hinselmann [79].

Early identification of CIN dramatically improved patient survival rates in the year 2020 [80]. Most cervical cancer detection algorithms rely on natural image object detection technologies, with only minor improvements made to account for the complex application scenario with respect to cervical lesion detection. The suggested method’s sensitivity at four false positives per image as well as average precision are enhanced by 2.79 and 7.2%, respectively, when compared to the baseline (Retinanet) [81]. Chen et al. (2020) first established the feasibility of using CT imaging and radiomics to create a low-cost image marker for detecting LN metastasis in cervical cancer patients. Here, the model was trained to utilize a leave-one-case-out (LOCO) cross-validation strategy with a total accuracy of 76.4%. Li et al. (2020) proposed a DL framework with regard to the accurate identification of LSIL+ (which includes CIN and cervical cancer) employing time-lapsed colposcopic images. All of the fusion methods that are compared perform better than the automated cervical cancer diagnosis systems that are currently in place and utilize a single time slot. The best fusion strategy was discovered to be a convolutional graph network with edge features (E-GCN). A novel framework built around a strong feature Convolutional Neural Networks (CNN)-Support Vector Machine (SVM) model was presented to properly categorize the cervical cells, according to research by Dongyao Jia et al. (2020). On two distinct datasets, the suggested technique was assessed using the metrics of accuracy (Acc), sensitivity (Sn), and specificity (Sp). The outcomes suggested that the CNN-SVM model with strong features might be utilized to classify cells for early cervical cancer screening [82].

A potential technique for the diagnosis of cervical cancer with parametrial infiltration is the combination of whole-tumor dynamic contrast-enhanced MRI and texture analysis [83]. Ktrans, energy, and entropy work more effectively together than separately, particularly when it comes to increasing diagnostic sensitivity. Fuzzy logic and adaptive neuro-fuzzy inference system (ANFIS) classification method-based cancer area detection and segmentation in cervical images were suggested by Ramasamy and Chinnasamy in 2020. Fuzzy logic is employed to identify the thick and thin edges, which are then combined using an image fusion approach at the pixel level. The suggested cervical cancer detection system has a classification rate average of 98.8%. In comparison to earlier suggested approaches for cervical cancer estimation, the CCPM result demonstrated more accuracy [84]. The sensitivity, specificity, and accuracy of the suggested cervical cancer segmentation methods presented in this paper are 98.1%, 99.4%, and 99.3%, respectively. A model for early cervical cancer prediction (CCPM) has been developed by researchers, utilizing risk indicators as inputs. In comparison to earlier suggested approaches for cervical cancer estimation, the CCPM results demonstrated more accuracy. For quick and effective action at the early stages of the disease, a mobile application that may gather information on cervical cancer risk factors and offer CCPM findings has been created [85].

Apart from that, [15] adopted a voting method that takes into account the issues with earlier research on cervical cancer. To assess the suggested procedure, several measures are implemented. According to the findings, the voting approach may be used to accurately forecast the chance of having cervical cancer. In comparison to previous techniques, the one that is being presented is more scalable and practical. The key finding by Singh and Goyal (2020) is the choice of the optimal ML algorithm with the maximum accuracy. Several algorithms were able to achieve up to 100%. Although a method such as LR with L1 regularization has a 100% accuracy rate, it consumes too much CPU time [16].

To effectively recognize the nucleus as well as the cytoplasm boundary of the Pap smear cell as a way to diagnose cervical cancer, an enhanced normalized graph cut with generalized data for enhanced segmentation (INGC-GDES) method was presented. In comparison to earlier methods, the suggested INGC-G DES mechanism leads to a 28% improvement in classification accuracy [13]. To the best of our knowledge, research has demonstrated the potential of Mueller matrix image processing as a unique strategy for the detection of cancer and precancer [86]. Sections of the human uterine cervix’s normal and precancerous tissue were utilized in the study. The research explained the creation of a DNA-based electrochemical biosensor that is sensitive and selective for the early detection of HPV-18. As a proprietary, accurate, sensitive, and quick diagnostic approach for HPV 18 in the polymerase chain reaction (PCR) of actual samples, the suggested biosensor can be presented. On a screen-printed carbon electrode (SPCE), a nanocomposite of reduced graphene oxide (rGO) as well as multiwalled carbon nanotubes (MWCNTs) was electrodeposited [87].

A study conducted by Rehman et al. (2020) reported that an auto-assisted cervical cancer screening system is suggested that utilizes a CNN trained on the Cervical Cells database. The system provides better performance than its previous counterparts under various testing conditions. For the 2-class problem, the classification accuracy of SR, SVM, and GEDT is determined to be 98.8%, 99.5%, and 99.6%, respectively [17]. Validation of Association Rule Mining using the Test Train Approach (VARMTTA), a data-driven methodology, was put out by Logeswaran et al. (2020). Employing the train-test validation approach lowers the number of rules that are generated from the dataset. This technique makes use of conventional measures, including sensitivity, precision, and total accuracy [14]. According to Sahoo et al. (2020), using a common path interferometric setup, low-coherence backscattered images of precancerous cervical tissue sections were recorded. These low-coherence images were subjected to a two-dimensional multifractal detrended fluctuation analysis (2D MFDFA) in order to examine the fluctuations in their fractal nature. The RI fluctuations showed long-range relationships, and multifractality was shown to be greater for cervical cancer with higher grades. It was discovered that normal and CIN-I, CIN-I and CIN-II, and normal and CIN-II had specificities and sensitivities of 94%, 88%, 93%, 96%, and 100%, respectively [88].

### 3.4. 2021–2022

B. Chitra and S. S. Kumar [89] reviewed the most recent soft computing techniques for detecting and classifying the most updated algorithms in current research. It is considered a literature review of the most common classification techniques for cervical cancer up to 2021. On top of that, Md. MamunAli et al. [90] employed clinical data for early cervical cancer detection. They applied a variety of data transformation techniques, such as Z-score, log, and sine functions, in addition to feature selection methods for specifying the most priority features for early detection of cervical cancer. Their results concluded that the logarithmic transformation feature is the best for biopsy data. On the other hand, sine is the best for cytology. However, the combination of sine as well as logarithmic is the best for the Hinselmann dataset, but for the Schiller dataset, the Z-score performance is the best. The classifiers utilized in this study are RF, Random Tree (RT), and instance-based nearest neighbor classifiers. For better performance, B. Chitra and S. S. Kumar [91] utilized the DL structure DesnNet 121 to classify Pap smear images. They apply various augmentation techniques to the dataset. The DL structure is optimized using the Mutation-based Atom Search Optimization (MASO) algorithm, which is employed to enhance the hyperparameters of DensNet121, for instance, the learning rate, the number of neurons in the dense layer, the number of epochs, patch size, and others. This approach obtains the best accuracy among existing techniques, which reaches 98.3%. Attempting other methods, such as recurrent neural networks, Zhang et al. [92] discussed the existing screening methods for cervical cancer that are based mainly on separated cells. Therefore, any misclassified cell causes poor accuracy. To overcome these limitations, they proposed a method that combines Long-Short Term Memory (LSTM) with a full CNN as well as fuzzy nonlinear regression. They exploited the time series method for improving cervical screening for cancer. Their procedure was accurate to 98.3%.

**Figure 3:**
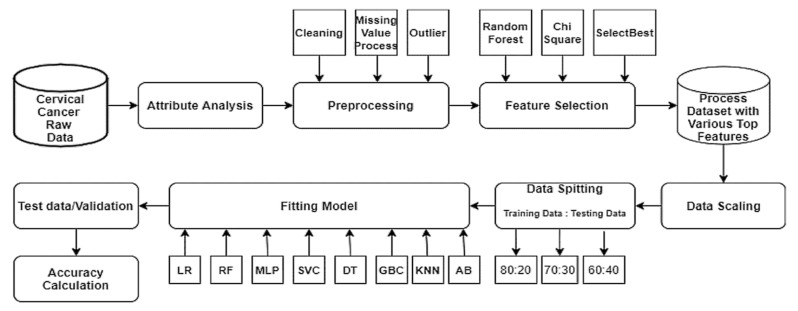
Automated invasive cervical cancer disease detection at an early stage via an appropriate ML model.

Sohely Jahan et al. [93] proposed an approach that is described in Figure 3. As it is clear, the raw cervical dataset is cured by outlier removal, cleaning methods, and excluding the records that have missing values. Various feature selection principles are utilized, for instance, Chi-square and RF, to find the most significant features. The selected features are scaled and split into 70:30 to train and test various types of classifiers such as Random Forest (RF), Logistic Regression (LR), Support Vector (SV), Multi-Layer Perceptron (MLP), Decision Tree (DT), Gradient Boosting (GB), K-nearest neighbour (KNN), and AdaBoost (AB) classifiers. MLP performed the best among all with a variety of features. On the other hand, all classifiers have almost the same high performance on 25 selected features.

**Figure 4:**
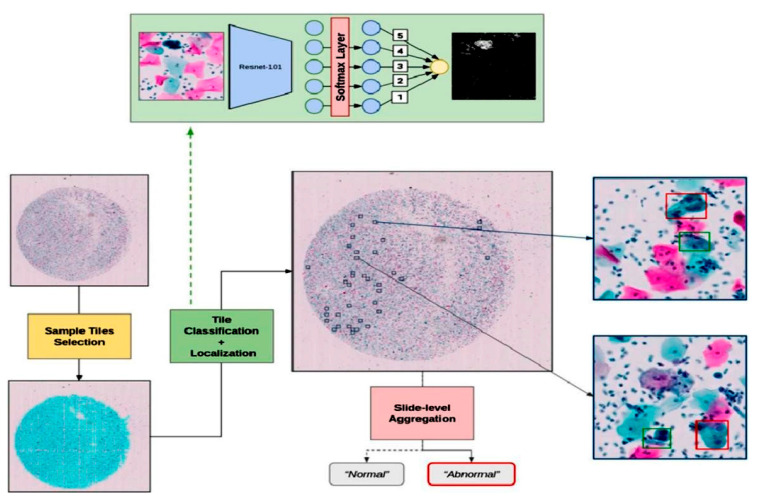
Graphical abstract of Antoine Pirovano et al.’s approach [95].

The research aims to improve accuracy with a reliable system. Therefore, Lei Cao et al. [94] suggested a more accurate system for detecting cervical cancer. Their method is based on a feature pyramid network to automatically classify cytological images by detecting abnormal cells. Their distinguished model has two features: the first is the reading way of the cervical cytology images, which is the same as pathologists, and the second is detecting abnormal cells at different scales using a multi-scale region-based fusion network. Their designed approach builds on clinical knowledge about abnormal cervical cells based on their shapes and sizes. The performance of their approach is better than the DL approach. Their highest accuracy was 95.8% on the independent dataset. Their process is accurate and quick, and their diagnosis time is 0.04 s per image, which is faster than pathologists’ diagnoses. For dealing with big-size images such as 1000 × 1000 pixels, Antoine Pirovano et al. [95] proposed the classification under regression constraints. Their experiment enhanced the sensitivity by up to 80% for localizing malignancy in whole slide images. The proposed approach can be integrated with the pathology laboratory system to improve prediction. Figure 4 illustrates their approach.

Some researchers used nanotechnology techniques, where Sakshi Pareek et al. [96] utilized nanotechnology to design an electrochemical biosensor that is sensitive and accurate for human papillomavirus infection (HPV-16) that causes cervical cancer. The designed biosensor is label-free for DNA. The proposed biosensor exhibits excellent sensitivity and stability. This is the core point in the HPV-16 analysis in medical diagnosis fields. On the other hand, Huiting Zhang et al. [97] employed Raman spectroscopy of pre-cancerous lesions for early cervical cancer detection. Their method depends on the Raman spectrum signal of the pre-cancerous cell, then utilizes partial least squares (PLS) with the Relife method for feature extraction from the signal. The selected features are passed to KNN and ELM classifiers. The novelty in their work is the feature fusion in the feature extraction phase. The classifier’s performance was enhanced using feature fusion, where KNN accuracy elevated from 88.17% to 93.55% using feature fusion and ELM from 90.81% to 93.51%.

AI is the challenge of many researchers, such as Sukumar Ponnusamy et al. [98], who combine the artificial neural network and fuzzy system interference (ANFIS) with a watershed algorithm to process, segment, and classify the Pap smear images. They exploited the fuzzy rules to classify abnormal images into their types. Their findings contrast with the existing approach, and it is feasible with high accuracy for classifying malignant cells into their corresponding classes. On top of that, Hongzhen Zhou et al. [99] analyzed the cervical tumor by automatic feature extraction using a deep belief network in contrast-enhanced ultrasonography images. Their goal postulated the effectiveness of intelligent cervical cancer diagnosis on chemotherapy. Their results are presented in terms of higher sensitivity and accuracy for the diagnosis system. Other researchers focused on the segmentation of affected parts of cervical cells using online machine learning (OLM), which was carried out by Asma Daly et al. [100], who segmented the cervical cells using the pelvic region in magnetic resonance imaging (MRI). They obtained high accuracy when they compared their results with existing segmentation techniques. Another type of ML is majority voting, which is based on utilizing a single classifier prediction and then an ensemble of them to vote the major, as proposed by Qazi Mudassar Ilyas et al. [101], who suggested using the ensemble classifier with majority voting of the output. Their ensemble consists of SVM, DT, RF, Naïve Bayes (NB), KNN, LR, J48 DT, and MLP. The best accuracy reached 94% when applied to different benchmark datasets. On the other hand, it utilized other types of classifiers, such as AB, XGBoost, and RF, with the Firefly algorithm as a feature reduction method in addition to SMOTE, which is utilized to deal with imbalance problems in the data. The four diagnostic data sets are exploited (Schiller, Hinselmann, Biopsy, and Cytology). The accuracy is enhanced in terms of reducing the number of selected features [102]. Due to state-of-the-art DL approaches, Khaled Mabrouk Amer Adweb et al. [103] discriminate between normal and pre-cancerous cervical cells using Leaky-RELU and PRELU in residual neural networks. The optimum accuracy reached 90.2% in Leaky-RELU and PRELU and 100% in colposcopy cervical images. On the other hand, Anant R. Bhatt et al. [104] discussed the shortcomings of all existing binary classification methods and conventional neural networks with respect to cervical cancer images. Therefore, they suggested a new approach to extracting features and classifying cervical cancer into multiclasses in a whole slide image (WSI) using ConvNet and a transfer learning strategy. They achieved 99.7% accuracy for multiclass classification in the SIPaKMed dataset. Other research focused on cervical cancer detection employing image processing methods such as Balaji, G. N., et al. [105], which utilized Boykov–Kolmogorov Graph Cuts as well as Cloud Model-based Synergy Integrated Segmentation algorithms for identifying the boundary for cytoplasm and nuclei in cervical Pap smear images. They approved that their methods enhanced the prognosis of cervical cancer by 14% over the traditional segmentation methods. Other studies employed template matching between the measured electrical impedance spectra of cervical cells and the spectra generated from a 3D model of finite elements for cancerous and non-cancerous cervical cells. The matching between spectra is expressed as a score to determine the high strength between the finite element model and the concourse and non-cancerous cells. This method can be effective for cervical cancer detection [106]. Some studies focused on the concomitant presence of miRNA-9-5p in cervical cancer, which was detected by RT-PCR. The experiment concluded that MiRNA-9-5p could be used as a biological marker for cervical cancer, which can be profitable in the inhibition track by inhibiting the CXCR4 gene and protein [107].

Some studies used the Lambert-Beer law to calculate the absorption peak. They found that the absorption is proportional to the cell concentration [108]. In contrast, other studies worked on both breast and cervical cancer together by employing DL [109]. Their work focused on utilizing the concepts of type of cancer, breast or cervical, whether it is located internally or externally, in addition to the imaging modality, whether it is mammography, ultrasound cytology, or colposcopy. Their results compared clinical diagnoses with DL. They conclude that DL can be an efficient tool for diagnosing cervical or breast cancer that can be replaced by clinician diagnosis. One Nobel and the most effective study depend on the fluorescence signal of urine samples [110]. They collected data using urine samples from 1500 patients and compared them with the healthy subjects, which formed control samples. They achieved a high true positive rate, reaching 74%. Their experiments can be conducted with simple requirements, such as fluorescence device analyses. With an amount of 200 μL, this process for diagnosis needs almost 40 min. On the other hand, the detection of affected papillomaviruses using photothermal-triggered multi-signal readout point-of-care testing (POCT). This bioassay method is realized and sensitive in linear ranges 10^−6^ ng/mL to 1 ng/mL with detection constraints reaching 1.60 × 10^−6^ ng/mL. This method is effective because it is fast, precise, and optimized for POCT. Therefore, it can be used in rural areas for the early detection of malignancy. Table 3 shows the reality of this method when it is compared with the available cervical cancer biomarker detection methods [111].

Combining texture features of the nucleus and cytoplasm in Pap smear images is a prominent tool to diagnose cervical cells. This method comes from the reality that doctors diagnose cervical cancer based mainly on the structure as well as the size of the cervical cells. Therefore, the Pap smear images in the Herlev dataset are segmented, and then the texture features are extracted to pass through a multilayer feed-forward neural network. The optimum results show high performance compared with the existing method [112]. On the other side, some studies employed DL and endomicroscopic images to diagnose CIN grade 2. The segmented nucleus is exploited to obtain relevant information for diagnosis. The dataset consisted of 1600 patients, and 20% were used for validation and testing. This approach results in sensitivity reaching 94% and specificity reaching 58%. Therefore, HPV infection test results are considered added features. The sensitivity remains at 94%, and the specificity is enhanced to 71% [113]. Apart from that, Dongyao Jia et al. [114] employed the YOLO (You Only Look Once) algorithm to detect abnormal cervical cells to guarantee the accuracy and rapidity of the model. This novel method forms a milestone for future work in automatic cervical cancer diagnosis.

Among the most prominent studies employed dual-tree complex wavelet transform (DTCWT) with a DL approach to classify Pap smear images into four categories: carcinoma in situ, normal, dysplastic, and superficial. The database is augmented for DL requirements using shearing and flipping transformations. The pixel conductivity of the augmented images is manipulated using multimodal (DTCWT). The CNN that has been used in their experiment is ResNet18, and they obtained a high accuracy of about 99% [115]. On the contrary, Chenjie Li et al. [116] assessed the effectiveness of 3D ultrasound imaging (TUI) on the local staging diagnosis of cervical cancer. Their suggestion is compared with existing methods such as pelvic examination and MRI. Their experiment was conducted on 35 cervical cancer patients, and the back-propagation algorithm was exploited to segment the images. Their results conclude that there is a high correlation between tumor size in MRI and THI, reaching 0.842, and that the correlation between MR and clinical examination reaches 0.654. This reveals high consistency between MR and THI and can be used for evaluating the local staging for cervical cancer.

For the combination of image processing and AI, most recent studies, such as AbuKhalil, T., et al. [117], enhanced Pap smear images using median filters and then segmented them using Outs thresholding techniques. The deep descriptors are extracted using ResNet and Inception modules. The resultant descriptors are passed to the recurrent neural network (RNN) to classify Pap smear images as cancerous or non-cancerous. In another study, Mohamed Ibrahim Waly et al. [118] used the Harvel data set to classify Pap smear images after applying preprocessing techniques such as a Gaussian filter to remove noise. Then identify the illness portion by segmenting the cell with the Tsallis entropy method with dragonfly optimization (TE-DFO). The segmented region is passed through the SqueezeNet model to extract automated graphical features. Weighted Extreme Learning Machine (ELM) is employed for cervix cell classification. On top of that, R. Elakkiya et al. [119] discussed the shortcomings of the existing methods for classifying cervical cell cancers. Mainly, they are based on accurate spotting and segmentation, in addition to handcrafted feature extraction. Therefore, they proposed Small-Object Detection-Generative Adversarial Networks (SOD-GAN) with a Fine-tuned Stacked Autoencoder (F-SAE) to detect the lesion faster and classify it into premalignant and malignant without segmentation and preprocessing. At the same time, M. Anousouya Devi et al. [120] utilized Neutrosophic Graph Cut-based for segmenting preprocessed Pap smear images into non-overlapping regions, which will lead to enhanced classification accuracy. This algorithm depends mainly on transforming preprocessed Pap smear images into the neutrophilic set. Then, the indeterminacy filter played a main role in integrating the intensity, including the spatial information of preprocessed images based on the indeterminacy value. This value specifies the weights for each pixel to define the graph. Finally, the maximum graph is determined to obtain the optimal segmentation results. This approach is better than existing detection methods by over 13%.

## 4. Discussion

Cervical cancer is a prominent health problem globally, with high mortality as well as incidence rates, particularly in developing countries [121,122]. Early detection is critical for the successful treatment and management of cervical cancer. The traditional method for cervical cancer screening is the Pap smear test, which involves the examination of cervical cells under a microscope for abnormalities. HPV is a very common sexually transmitted infection, with estimates estimating that up to 80% of sexually active women will become infected with HPV at some point in their lives. However, the majority of these infections will clear up on their own without causing any long-term health problems. There are many different types of HPV, and some types are more likely to cause cancer than others. However, this method is subjective and may miss precancerous lesions, leading to false negatives and a delayed diagnosis. Therefore, there has been further interest in establishing CAD methods to improve cervical cancer screening. CAD technology for cervical cancer detection has been extensively examined over the past few decades [123,124]. Between 1996 and 2022, significant advancements have been made in this field, leading to improved accuracy, sensitivity, and specificity of CAD methods. Early CAD systems utilized image processing and pattern recognition techniques to analyze digital images of cervical cells with the aim of identifying abnormal cells and lesions. However, these early systems had limited success due to low sensitivity and specificity.

In the early 2000s, ML algorithms were introduced to the field of CAD for cervical cancer detection. ML algorithms can analyze large datasets and learn from them to identify patterns and make predictions. This allowed for more accurate and automated analysis of digital images of cervical cells. ML-based CAD systems have shown promise in several studies, with improved sensitivity and specificity reported compared to traditional screening methods [125,126,127]. Among the most promising CAD systems for cervical cancer detection is the Hybrid Intelligent System for Cervical Cancer Diagnosis (HISCCD), which was developed in 2012. HISCCD is a combination of ML algorithms and rule-based systems that analyze digital images of cervical cells to detect abnormal cells and lesions. Several studies have reported improved sensitivity and specificity of HISCCD compared to traditional screening methods. Another promising CAD system is the Automated Cervical Screening System (ACSS), which was introduced in 2016. ACSS uses an ML-based algorithm to analyze digital images of cervical cells and identify abnormal cells and lesions. In a study comparing ACSS to the Pap smear test, ACSS showed higher specificity and sensitivity for detecting high-grade cervical intraepithelial neoplasia. In addition to these systems, there have been several other CAD systems developed over the years, each with its own strengths and limitations. One of the major challenges with CAD systems for cervical cancer detection is the lack of standardized protocols and data sharing, which limits their widespread adoption and validation.

The previous studies describe the most updated state-of-the-art techniques that were suggested, validated, and evaluated for early cervical cancer detection. Most researchers conducted their experiments utilizing image processing in addition to ML and DL. The pre-processing techniques are employed to enhance the visualization of Pap smear images and make feature extraction an easy and more accurate task. Other researchers skipped this step by utilizing DL techniques to extract features automatically, which reduces time and gives accurate results because all of the features excreted in this step are relevant to the corresponding class. However, many researchers focused on HPV, which plays the main role in the infection of cervical cancer. They focused on the nanotechnology track by designing a biosensor that can detect the infection and is distinguished by its stability and linearity. Other researchers focus on building a finite element model for both cancerous and noncancerous cells to study the electrical impedance spectroscopy and compare it with the tested cell to find the matching score between them. They count it as an alternative method that is more accurate than using a Pap smear screening test. Chemical reactions are also considered by other researchers by studying the fluorescence signals from the urine of the infected women and comparing those signals with those of healthy women.

Various methods have been carried out in this area, either in biochemistry, image processing, DL, signals, or nanotechnology tracks, to enhance and reach a highly accurate approach to diagnosing cervical cancer in its early stages. This will reduce the mortality rate among women and increase the chance of survival. In conclusion, CAD technology for cervical cancer detection has come a long way since its introduction in the 1990s. ML-based algorithms have shown promise in improving the accuracy and sensitivity of CAD systems for cervical cancer detection. HISCCD and ACSS are two of the most promising CAD systems, but extensive research and validation are required before they can be broadly applied.

## 5. Conclusions

Cervical cancer is a substantial public health issue globally, with more than half a million new cases and a quarter of a million deaths each year. Early detection and treatment of cervical cancer can significantly improve outcomes and save lives. Fortunately, there are several different methods for cervical cancer detection, each with its own limitations and advantages. The Pap smear test is the most broadly employed and popular technique with respect to cervical cancer detection. It is a low-cost, simple, and efficient way to screen for precancerous or cancerous changes in the cervix. The Pap smear test has undergone several improvements over the years, including the use of liquid-based cytology, which has improved its accuracy and sensitivity. However, the Pap smear test is not foolproof and can miss some cases of cervical cancer, especially in its early stages.

The recommended screening guidelines may vary depending on age, risk factors, and previous screening results. In developed countries, the adoption of cervical cancer screening programs has led to a significant decrease in cervical cancer mortality rates. However, in low- and middle-income countries, the lack of access to screening programs and cost-effective screening methods and vaccines is a significant barrier to early detection and effective treatment. Therefore, the development of simple, low-cost, and accurate screening methods that can be implemented in low-resource settings is essential. In recent years, machine learning (ML) and deep learning (DL) algorithms have been deployed to aid in cervical cancer diagnosis and treatment by identifying abnormal and normal cells automatically, precisely, and quickly. These algorithms have demonstrated high sensitivity and specificity in detecting abnormal cervical cells, indicating their potential use as an adjunct to traditional screening methods. However, more research is needed to evaluate the feasibility and effectiveness of these algorithms in real-world clinical settings.

In the future, the identification of important risk factors as well as the utilization of various segmentation pre-processing techniques can enhance the effectiveness of cervical cancer diagnosis and treatment. Bigger and more balanced data can also improve the performance of future classification systems. In conclusion, cervical cancer detection has come a long way over the years, with several different methods available, each with its advantages and limitations. The Pap smear test remains the most frequently employed method, but newer methods, including HPV testing, VIA, and VILI, are becoming more widely used. A colposcopy is also an important tool for follow-up and diagnostic purposes. Regular cervical cancer screening is critical for early detection and successful treatment. Women should discuss their screening options with their healthcare provider and follow the recommended guidelines for cervical cancer screening. By working together, we can continue to improve cervical cancer detection and save lives. Nevertheless, continued innovation and collaboration in this field may facilitate the enhancement of cervical cancer detection and ultimately lower the disease’s burden on women worldwide.

## Figures and Tables

**Table 1:** The specification for primary data searching.

Keyword	Cervix, Cervical, Cancer, Tumor, Detect, Diagnosis
Inclusion	Article, Journal, English, computer science, and engineering
Exclusion	Pure medicine, review article, other languages
Final Search String (Scopus)	TITLE ((cervix OR cervical) AND (cancer OR tumor) AND (detect* OR diagnosis)) AND (LIMIT-TO (PUBSTAGE, “final”)) AND (LIMIT-TO (DOCTYPE, “ar”)) AND (LIMIT-TO (SUBJAREA, “ENGI”) OR LIMIT-TO (SUBJAREA, “COMP”)) AND (LIMIT-TO (LANGUAGE, “English”)) AND (LIMIT-TO (SRCTYPE, “j”))
Number of Primary Article	108

**Table 2:** Pap smear image classification in the Herlev dataset [9].

Cell	Class Name	Cell Count	Sub-Total
Normal	Normal Superficial Squamous	74	242
	Normal Intermediate Squamous	70	
	Normal Columnar	98	
Abnormal	Carcinoma In Situ	150	675
	Light Dysplastic	182	
	Moderate Dysplastic	146	
	Severe Dysplastic	197	
	Total	917	917

**Table 3:** Comparison with multiple techniques with regard to cervical cancer biomarker detection.

Detection Methods	Targets	Liner Range	LOD (Limit of Detection)
Magnetic sensor	VCP	25–200 ng/mL	2.5 × 10^−5^ ng/mL
Colorimetric assay	HPV	20–2500 nM	1.03 nM
Electrochemical	pGEM-T/E6	40–5000 ng/mL	0.016 ng/mL
Electrochemical	GST-p16	15.6–250 ng/mL	1.3 ng/mL
Swab immunoassay	E6 protein	10^−6^–1 ng/mL	1.60 × 10^−6^ ng/mL

## Data Availability

Not applicable.
